# Differential distribution of HP1 proteins after trichostatin a treatment influences chromosomal stability in HCT116 and WI-38 cells

**DOI:** 10.1186/s13008-014-0006-2

**Published:** 2014-12-30

**Authors:** Rodrigo González-Barrios, Ernesto Soto-Reyes, Ricardo Quiroz-Baez, Eunice Fabián-Morales, José Díaz-Chávez, Victor del Castillo, Julia Mendoza, Alejandro López-Saavedra, Clementina Castro, Luis A Herrera

**Affiliations:** Unidad de Investigación Biomédica en Cáncer, Instituto Nacional de Cancerología (INCan)-Instituto de Investigaciones Biomédicas (IIB), Universidad Nacional Autónoma de México (UNAM), México, DF 14080 México; Departamento de Investigación Básica, Dirección de Investigación, Instituto Nacional de Geriatría, Secretaría de Salud, México, DF 10200 México; Instituto de Investigaciones Biomédicas, Universidad Nacional Autónoma de México, Circuito Escolar S/N, Ciudad Universitaria, Coyoacán, México, DF 04510 México

**Keywords:** HP1, Centromeric chromatin, TSA, Chromosome instability, CENP-A

## Abstract

**Background:**

Heterochromatin protein 1 (HP1) is important in the establishment, propagation, and maintenance of constitutive heterochromatin, especially at the pericentromeric region. HP1 might participate in recruiting and directing Mis12 to the centromere during interphase, and HP1 disruption or abrogation might lead to the loss of Mis12 incorporation into the kinetochore. Therefore, the centromere structure and kinetochore relaxation that are promoted in the absence of Mis12 could further induce chromosome instability (CIN) by reducing the capacity of the kinetochore to anchor microtubules. The aim of this study was to determine whether alterations in the localization of HP1 proteins induced by trichostatin A (TSA) modify Mis12 and Centromere Protein A (CENP-A) recruitment to the centromere and whether changes in the expression of HP1 proteins and H3K9 methylation at centromeric chromatin increase CIN in HCT116 and WI-38 cells.

**Methods:**

HCT116 and WI-38 cells were cultured and treated with TSA to evaluate CIN after 24 and 48 h of exposure. Immunofluorescence, Western blot, ChIP, and RT-PCR assays were performed in both cell lines to evaluate the localization and abundance of HP1α/β, Mis12, and CENP-A and to evaluate chromatin modifications during interphase and mitosis, as well as after 24 and 48 h of TSA treatment.

**Results:**

Our results show that the TSA-induced reduction in heterochromatic histone marks on centromeric chromatin reduced HP1 at the centromere in the non-tumoral WI-38 cells and that this reduction was associated with cell cycle arrest and CIN. However, in HCT116 cells, HP1 proteins, together with MIS12 and CENP-A, relocated to centromeric chromatin in response to TSA treatment, even after H3K9me3 depletion in the centromeric nucleosomes. The enrichment of HP1 and the loss of H3K9me3 were associated with an increase in CIN, suggesting a response mechanism at centromeric and pericentromeric chromatin that augments the presence of HP1 proteins in those regions, possibly ensuring chromosome segregation despite serious CIN. Our results provide new insight into the epigenetic landscape of centromeric chromatin and the role of HP1 proteins in CIN.

**Electronic supplementary material:**

The online version of this article (doi:10.1186/s13008-014-0006-2) contains supplementary material, which is available to authorized users.

## Background

Heterochromatin protein 1 (HP1) binds to histone H3 proteins that have been methylated at lysine 9 by SUV39H1, thereby propagating the methylation along chromatin [1]. HP1 function is highly important for the establishment, propagation, and maintenance of constitutive heterochromatin [2], especially at the pericentromeric region, which is enriched with H3K9me3 and H4K20me3 marks, hypoacetylated H3 and H4, and highly methylated regions along most of its satellite repeats [3,4]. Due to its juxtaposition with centromeric chromatin, it has been suggested that the organization and stability of the pericentromeric region are crucial for correct chromosomal segregation during mitosis; therefore, this region is important for genome stability [3,5].

HP1 also plays a role in centromeric sister chromatid cohesion [6], telomere maintenance, and DNA repair [7]. In humans, these functions are performed in a specific manner by each of the three identified HP1 subtypes: HP1α, HP1β, and HP1γ [8,9]. HP1 protein localization differs in the interphase nucleus, with HP1α typically found in pericentric and telomeric chromatin and HP1β normally found in heterochromatin regions [10].

Live cell microscopy analyses have demonstrated that the localizations of human HP1α and HP1β have specific functions at different points of the cell cycle. An exchange between human HP1α and HP1β has been observed at centromeric heterochromatin during mitosis [10]. This exchange is mediated by differences in the chromoshadow domain sequences of these proteins [10]. Increasing evidence has shown that the Knl1-Mis12-Ndc80 (KMN) protein complex is a binding partner of HP1 in humans, in which HP1 might participate in recruiting and directing Mis12, a kinetochore complex component that is a subunit of the KMN network that resides at the centromere during interphase and stably associates with the kinetochore during mitosis [11-13]. The disruption or abrogation of HP1 is believed to lead to tumor formation, and the absence of HP1 might also lead to the loss of Mis12 incorporation into the kinetochore. Therefore, the centromere structure and kinetochore relaxation that are promoted by the absence of Mis12 could further induce chromosome instability (CIN) by reducing the capacity of the kinetochore to anchor microtubules [14].

These findings suggest that the deregulation of epigenetic components in the kinetochore complex could result in chromosomal defects and CIN development. Furthermore, it has become increasingly clear that chromatin composition affects centromere determination and establishment. Nevertheless, the genomic and chromatin modifications that are necessary to establish and maintain the centromere remain unknown. It has been suggested that the DNA sequence alone is not always sufficient for centromere establishment or function [15], supporting theories that postulate the involvement of epigenetic mechanisms [16]. Thus, the aim of our study was to determine whether alterations in the localization of HP1 proteins modify Mis12 recruitment to the centromere and whether changes in the expression of HP1 proteins and H3K9 methylation at centromeric chromatin lead to an increase in CIN.

To address these questions, we evaluated HP1 proteins during the cell cycle. In addition, we treated cells with trichostatin A (TSA), an inhibitor of histone deacetylase (HDAC) enzymes, to indirectly antagonize centromeric heterochromatin and HP1 binding by reducing H3K9me3 abundance. Our results show that the TSA-induced reduction in heterochromatic histone modification of centromeric chromatin reduced HP1 levels at the centromere in WI-38 cells and that this reduction was associated with cell cycle arrest and CIN. However, in HCT116 cells, HP1 proteins relocated to centromeric chromatin in response to TSA treatment, and this re-localization was induced even after H3K9me3 was reduced in the centromeric nucleosomes. The enrichment of HP1 proteins in HCT116 cells was associated with increased CIN, suggesting a response mechanism in the centromeric and pericentromeric chromatin that augments the presence of HP1 proteins in those regions, possibly ensuring chromosome segregation despite serious CIN.

## Results

### Chromosome instability is induced by TSA

HP1 proteins and H3K9me3 have been shown to play an important role in chromosome stability. There are several reports on the different types of CIN promoted by TSA treatment in a wide range of concentrations and periods of exposure [17-19]. Therefore, we evaluated if treatments with TSA promoted a similar effect in the induction of CIN in WI-38 and HCT116 cells.

**Figure 1:**
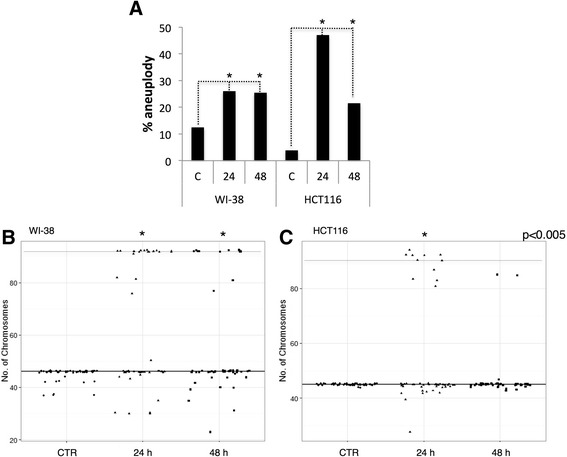
**Trichostatin A (TSA) treatment generates chromosome instability primarily in HCT116 cells.** Chromosome counting was performed after cells were treated with 1 μM TSA for 24 and 48 h. **(A)** The percentage of aneuploidy was greater than 26% after the 24 and 48 h TSA treatments in WI-38 cells, and the effect of TSA was more pronounced in HCT116 cells after 24 h (at 47%) but decreased to 21% after 48 h of exposure. **(B-C)** The representation of the number of chromosomes from the controls and the 24- and 48-h TSA-treated WI-38 **(B)** and HCT116 cells **(C)**, showing gains and losses after counting; the black line designates the 2n cells, and the dotted line designates the 4n cells. The total number of chromosomes in 50 cells was counted. The Kruskal-Wallis test yielded p < 0.05 compared with the values of the control (CTR).

TSA induced aneuploidy in both cell lines (Figure 1A). After TSA treatment for 24 h, 26% of WI-38 cells were aneuploid, and this frequency was maintained for at least 48 h post-treatment. In contrast, 47% of HCT116 cells were aneuploid after TSA treatment for 24 h; however, this frequency was lower (22%) after treatment for 48 h. WI-38 cells lost more than 6 chromosomes or gained more than 20 chromosomes (Figure 1B). A high number of HCT116 cells were aneuploid after 24 h of treatment; however, after 48 h, the rate of chromosomal gains and losses was reduced (Figure 1C, Table 1). After TSA treatment for 24 h, 32% of WI-38 cells were 4n; after treatment for 48 h, 19.6% of the cells remained 4n, indicating that WI-38 cells could not properly segregate following TSA treatment (Table 1). Only 4% of HCT116 cells were 4n after treatment for 24 h, and no 4n cells were found after 48 h (Table 1).

**Table 1:** **Analysis of total chromosome number in each cell after 24 and 48 h of trichostatin A (TSA) treatment**

	**WI-38**			**HCT116**		
	**Control**	24 h*	48 h*^,^**	**Control**	**24 h**	48 h**
**Mode**	42 (87.5%)	21(42%)	28 (54.9%)	49 (96.08%)	25 (54.9%)*	40 (78.43%)
**Loss**	6 (12.5%)	8 (16%)	11 (21.56%)	2 (3.92%)	14 (27.5%)*	7 (13.73%)
**Gain**	0	5 (10%)	2 (3.92%)	0	10 (19.6%)*	4 (7.84%)
**4n**	0	16 (32%)	10 (19.6%)	0	2 (3.9%)	0
**Total**	**48**	**50**	**51**	**51**	**51**	**51**

Loss was considered below 2n, and gain was considered above 2n.

*Levene’s test p < 0.001; treatment versus control.

**Levene’s test p < 0.05; 24-h treatment versus 48-h treatment.

### Centromeric chromatin dynamics during the cell cycle

**Figure 2:**
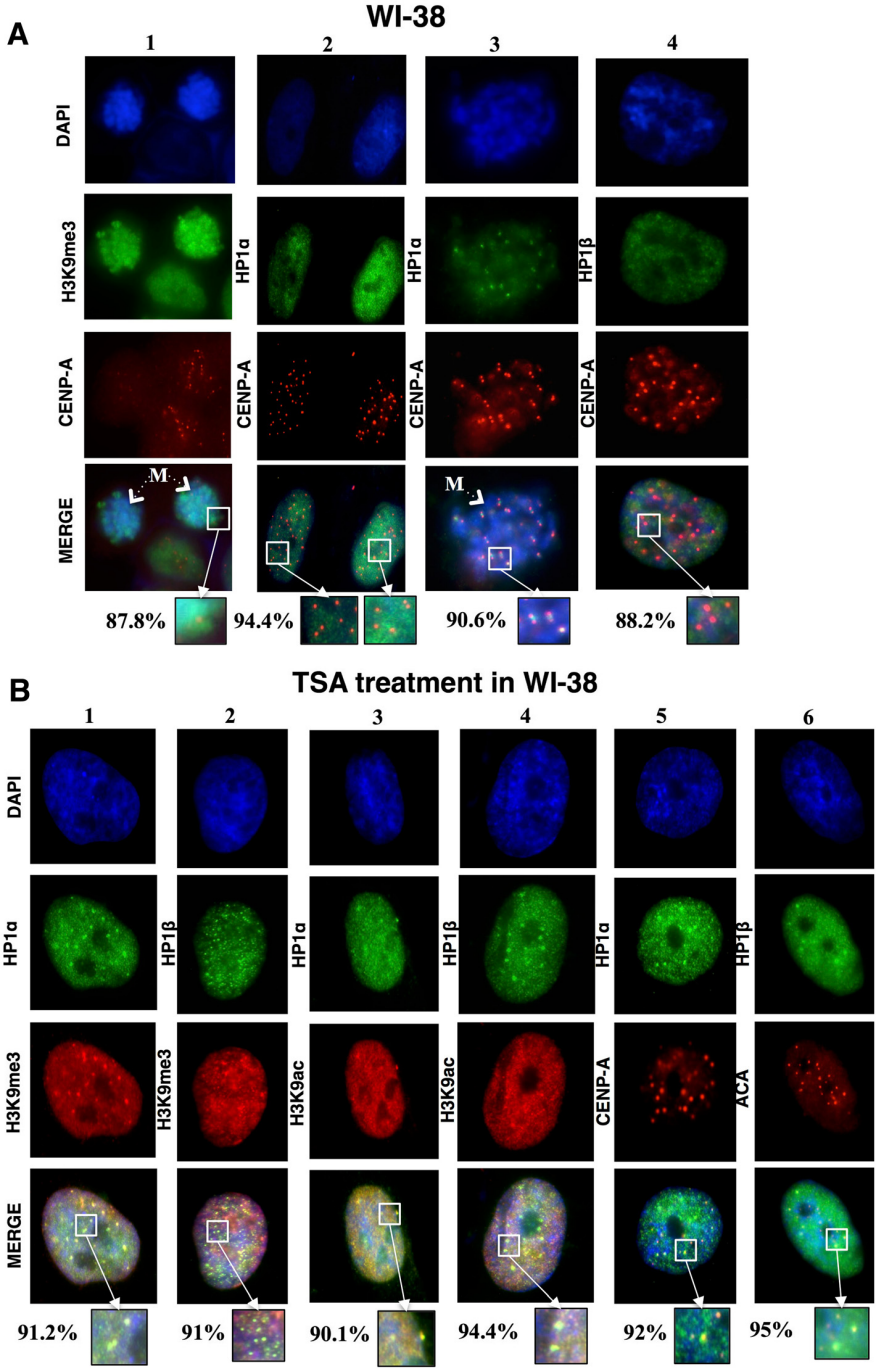
**Centromeric localization of HP1α and HP1β in WI-38 cells under basal conditions and after TSA treatment. (A)** WI-38 cell fluorescent microscopy localization of CENP-A with H3K9me3 (lane 1), HP1α (lane 2-3) and HP1β (lane 4). **(B)** Chromatin localization by fluorescent microscopy in WI-38 cells after TSA treatment comparing H3K9me3 with HP1α (lane 1) or HP1β (lane 2) H3K9ac with HP1α (lane 3) or HP1β (lane 4), the centromeric localization of HP1α compared with CENP-A (lane 5), and HP1β compared with ACA (lane 6). The DNA is marked with DAPI; the images show the most common distribution of proteins after the analysis of 100 cells (%); the boxes represent a magnification of the immunofluorescence results; M, mitotic cell.

**Figure 3:**
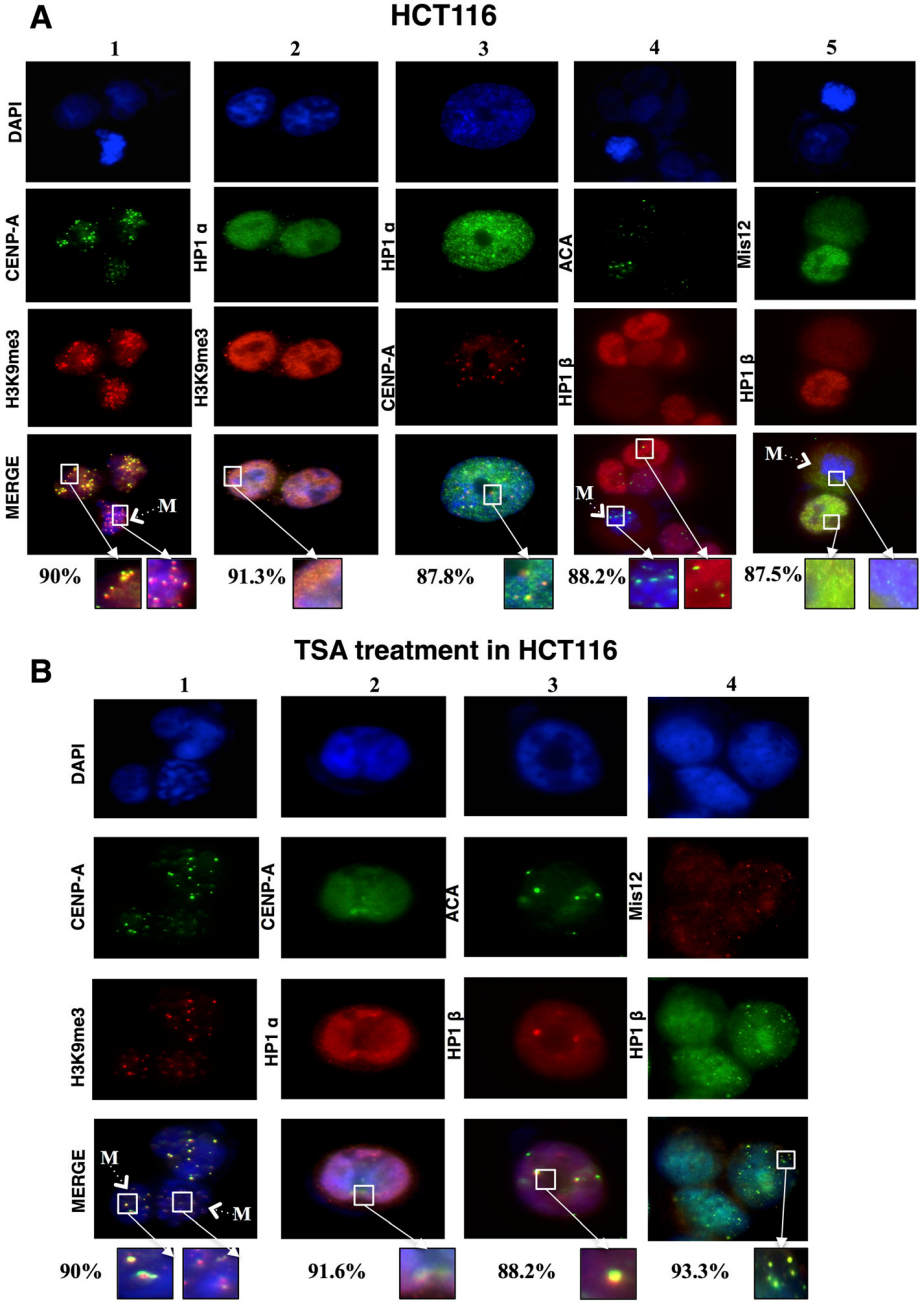
**Centromeric localization of histone marks, HP1α, and HP1β in HCT116 cells under basal conditions and after TSA treatment. (A)** HCT116 chromatin localization by fluorescent microscopy of CENP-A with H3K9me3 (lane 1) or HP1α (lane 2), H3K9me3 and HP1α (lane 3), ACA and HP1β (lane 4), and HP1β and Mis12 (lane 5). **(B)** Centromeric localization of H3K9me3 and CENP-A (lane 1), CENP-A and HP1α (lane 2), ACA and HP1β (lane 3) and Mis12 co-localization with HP1β (lane 4). The DNA is marked with DAPI; the images show the most common distribution of the proteins after the analysis of 100 cells (%); the boxes represent a magnification of the immunofluorescent results; M, mitotic cell.

To observe the localization of HP1α and HP1β proteins throughout the cell cycle, as well as their association with H3K9me3 and CENP-A, we performed immunofluorescence assays in WI-38 (Figure 2A) and HCT116 (Figure 3A) cells. In WI-38 cells, we explored the nuclear localization of H3K9me3 and CENP-A, both of which were enriched at centromeric loci and neighboring regions. This enrichment persisted in mitotic cells (Figure 2A). Because H3K9me3 is the epigenetic modification that is recognized by the HP1 protein chromodomain, and given the importance of HP1 proteins for proper chromosome alignment and mitotic progression [11,19], we evaluated the nuclear localization of the HP1α and HP1β isoforms together with CENP-A. We observed little difference in the localization of both HP1 isoforms at the centromere. HP1α was localized to regions neighboring CENP-A, which are likely pericentromeric heterochromatin, and also occupied other chromatin regions. HP1β showed a similar localization pattern (Figure 2A). Therefore, although both isoforms play a critical role in establishing and maintaining heterochromatin, they might play different roles in terms of the surrounding centromeric chromatin.

We then determined whether the dynamics and localization of H3K9me3, HP1α, and HP1β are conserved in a cancer cell line with no CIN and with stable chromosome segregation such as HCT116 cells. For this purpose, we performed immunofluorescence microscopy assays to observe the centromeric localization of HP1. H3K9me3 co-localized with CENP-A during interphase; during mitosis, it either co-localized or was enriched in the region neighboring the centromere (Figure 3A). As expected, the HP1 isoforms showed similar patterns of localization in both cell lines, suggesting conserved chromatin behavior for both H3K9me3 and HP1 (Figure 3A). We observed almost no co-localization of the euchromatic marks H3K9ac and H3K4me2 with CENP-A, indicating that open chromatin marks are present at centromeric regions at a low frequency in both cell lines (Additional file 1: Figure S1A-B). Considering that HP1 has been associated with the Mis12 complex, we observed that HP1 co-localizes with Mis12 during interphase, but this localization changes slightly during mitosis (Figure 3A).

To confirm the presence of HP1 at centromeric chromatin and to assess its dynamics during interphase and mitosis, we selected HCT116 cells with stable chromosomal segregation. We treated these cells with nocodazole for 12 h and isolated the mitotic cells using the shake-off method. We then performed a ChIP assay for activating (H3K4me2 and H3K9ac) and repressive (H3K9me2/me3) histone marks, as well as for CENP-A, Mis12, HP1α, and HP1β, in mitotic and interphase cells. As controls for open and closed chromatin, we evaluated the GAPDH and WIF1 promoter regions, respectively. The GAPDH promoter, as expected, was enriched with the markers of gene activation H3K4me2 and H3K9ac; the abundance of these marks was increased during mitosis, but the increase was proportional to the gain in total H3 in the region (Additional file 2: Figure S2A). We used the WIF1 gene promoter region as a positive control for gene silencing due to its role as a WNT inhibitor; this promoter is known to be enriched with H3K9me2 and H3K9me3 modifications after cell differentiation during embryonic development. We found that this region was enriched with repressive marks and that these marks were increased during mitosis (Additional file 2: Figure S2B).

Because centromeric chromatin has been poorly studied by ChIP analysis and because there have been contrasting results in different models, we designed primers for global satellite-α repeats and analyzed the 171-bp monomer sequence. Our results showed that H3K9me2 and HP1β are present during interphase and mitosis, whereas the presence of H3K4me2, H3K9ac and H3K9me3 histone marks fluctuate throughout the cell cycle (Additional file 2: Figure S2C).

**Figure 4:**
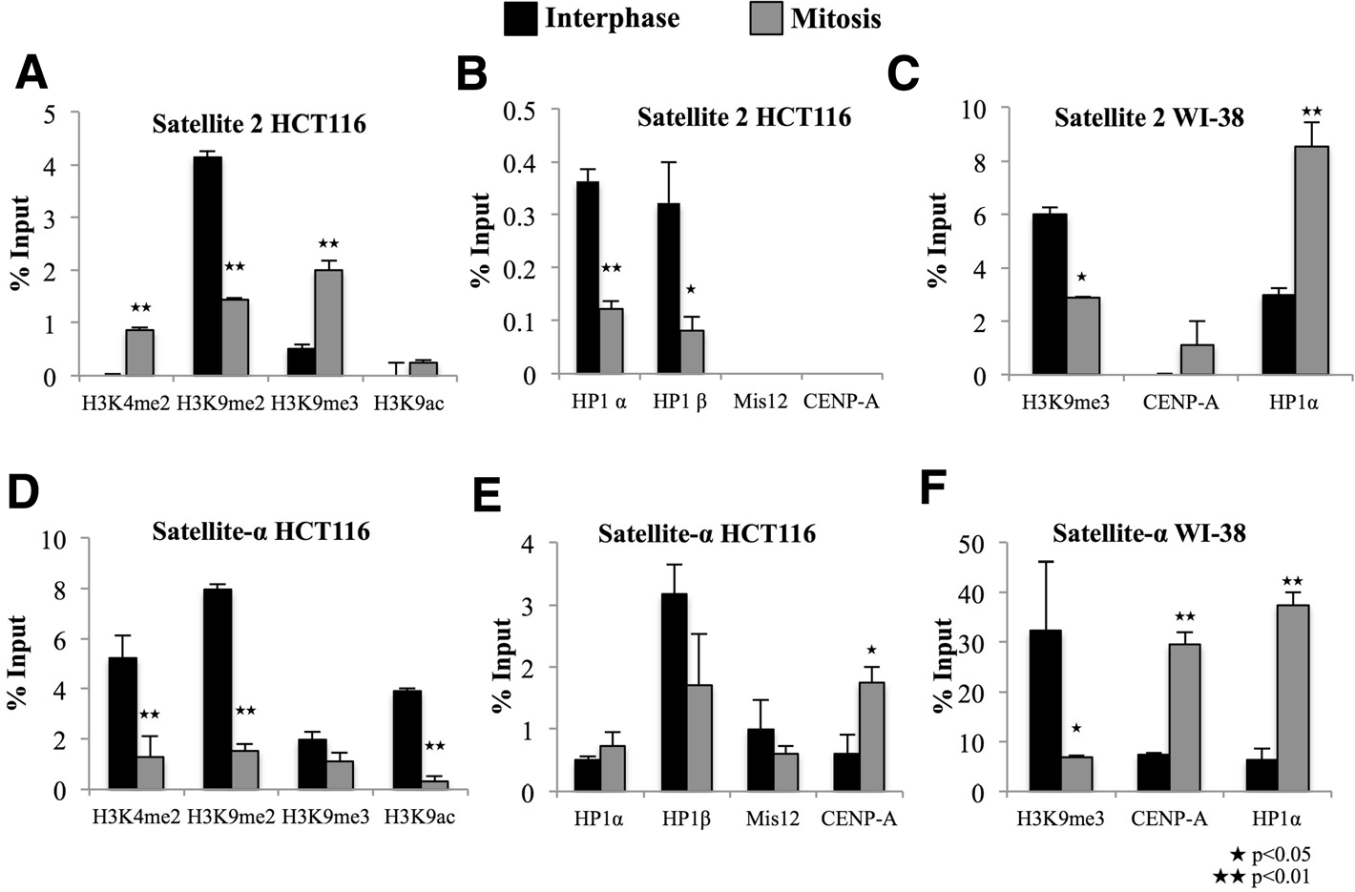
**Epigenetic changes and protein dynamics at satellite-2 and satellite-α regions during interphase and mitosis in HCT116 and WI-38 cells.** qRT-PCR analysis of the satellite-2 **(A-C)** and satellite-α **(D-F)** repeats was performed on DNA obtained from anti-H3K4me2, H3K9me2, H3K9me3, H3K9ac, HP1α, HP1β, Mis12 and CENP-A ChIP assays in interphase (black) and mitotic (gray) cells. Normal rabbit IgG was employed as a negative control. ★p < 0.05 and ★★p < 0.01 represent significant differences between interphase and mitosis, as evaluated by Student’s T-test.

However, the satellite-α repeat arrangement varies at the centromere, and CENP-A/H3 nucleosomes are scattered thorough the centromeric sequence. We therefore designed primers that were specific for satellite-α and satellite-2 regions of chromosome 1 to confine the analysis of chromatin changes to these regions. We evaluated chromosome 1 satellite-2 pericentromeric regions, which were enriched with H3K9me2 and H3K9me3 during interphase; as expected, HP1α and HP1β were also present during this phase (Figure 4A-B) because satellite-2 is a well-known heterochromatic region. Interestingly, we observed a 2-fold enrichment of H3K4me2 and a 50% reduction of H3K9me3 in mitotic cells. We did not observe Mis12 and CENP-A at the satellite-2 repeat. We then questioned whether the same modulation occurred in normal cells such as WI-38 cells. We explored the same satellite-2 regions during interphase and mitosis and found that in interphase cells, H3K9me3 was abundant alongside HP1α, which is typical of the pericentromeric constitutive heterochromatin domain (Figure 4C). During mitosis, H3K9me3 was reduced, but HP1α was heavily enriched, suggesting a role for HP1α at pericentromeric heterochromatin during chromosome segregation (Figure 4C). We observed an unexpected enrichment of CENP-A at satellite-2 chromatin during mitosis (Figure 4C).

Moreover, in the chromosome 1 satellite-α repeat region, a mixed histone epigenetic landscape was found, in which active and repressive histone marks were present throughout the cell cycle (Figure 4D-E). We observed a significant enrichment of CENP-A during mitosis, whereas the enrichment of known H3 modifications was reduced (Figure 4D). The satellite-α region in WI-38 cells was also enriched with CENP-A and HP1α. Although H3K9me3 was reduced during mitosis, it remained present at the centromere (Figure 4F), suggesting that HP1α plays a different role at the centromere in this cell line than in HCT116 cells. In contrast to the immunofluorescence results, we detected HP1α and HP1β in the specific satellite-α region, and their enrichment fluctuated slightly during mitosis (Figure 4E-F).

### TSA treatment causes HP1 proteins to re-localize to centromeric chromatin in HCT116 but not WI-38 cells

To observe the effect of antagonizing heterochromatic regions of pericentromeric and centromeric chromatin, we treated WI-38 cells with 1 μM TSA, which leads to chromatin relaxation and gene expression modulation [20,21]. To evaluate the effect of TSA on the cell cycle, cells were treated for 24 and 48 h; the drug was reintroduced via fresh medium every 24 h. The effect of this treatment on protein nuclear localization was observed by fluorescence microscopy. TSA reduced H3K9me3 levels, as expected, and also reduced the protein levels of HP1α and HP1β (Figures 2A and 3B). Clear foci of H3K9me3 remained after treatment, and these foci co-localized with the HP1α and HP1β isoforms (Figures 2B and 3B). We also observed that these HP1 protein foci localized to the same regions occupied by CENP-A, suggesting that both H3K9me3 and HP1α/β are more enriched in the centromeric region, as defined by the localization of CENP-A and ACA. These results suggest that H3K9me3 was preserved at the centromere and that both HP1 proteins accumulate at centromeric chromatin in response to TSA treatment (Figures 2B and 3B). When treated with TSA, both HCT116 and WI-38 cells presented H3K9me3 at CENP-A foci, which were also occupied by both HP1 isoforms (Figures 2B and 3B).

Considering that HP1 has been associated with the Mis12 complex, we determined whether Mis12 localization was affected by TSA. Interestingly, Mis12 localization showed a strong correlation with HP1β localization and was also enriched at TSA-promoted HP1β foci, suggesting that this kinetochore foundation protein is associated with HP1β not only during mitosis but also during interphase (Figure 3B).

**Figure 5:**
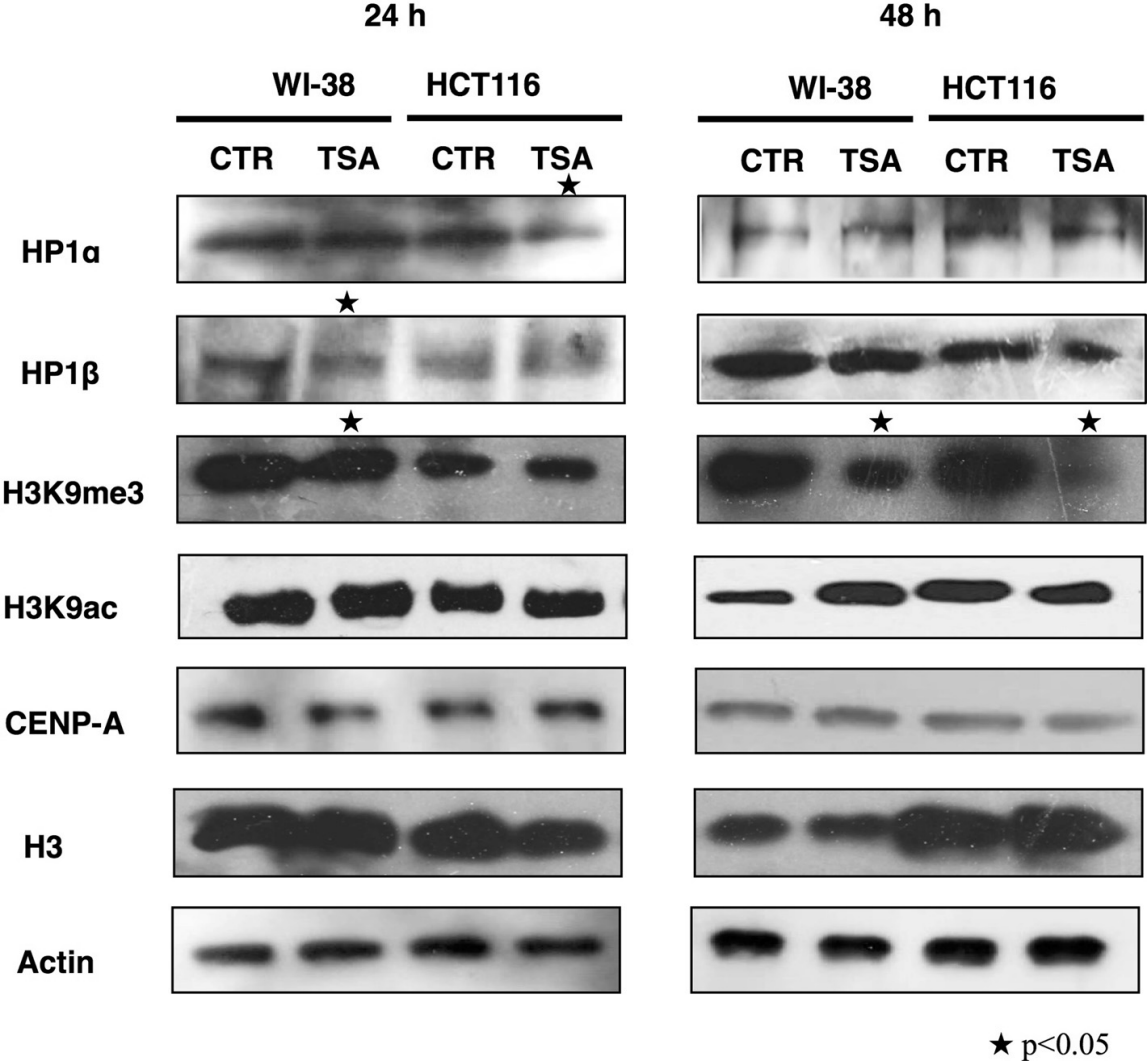
**The protein content in HCT116 and WI-38 cells treated with TSA.** Representative Western blot of HP1α, HP1β, H3, H3K9me3, H3K9ac and CENP-A levels after treatment with 1 μM TSA for 24 or 48 h. The experiments were conducted in 3 independent determinations that were performed in duplicate for each experimental condition; the asterisk indicates p < 0.05 compared with the value of the control (CTR), as obtained by Student’s t-test.

To determine whether these changes in HP1 protein localization were related to alterations in total protein levels, we performed an immunoblot assay in both cell types before and after the TSA treatments for 24 and 48 h. After 24 h, we observed a significant reduction in HP1α in HCT116 cells only; no significant changes were found in WI-38 cells. After 24 h of TSA exposure, HP1β was decreased in WI-38 cells; however, the original levels were restored after 48 h. No changes were observed in the abundance of HP1β in HCT116 cells or the levels of CENP-A in either cell line after TSA treatment (Figure 5). Moreover, we found no significant changes in H3K9ac levels after TSA treatment, although we did observe a tendency to accumulate acetylation after 48 h of treatment in WI-38 cells. Nevertheless, H3K9me3 levels were significantly decreased, especially after 48 h of TSA exposure, suggesting not only that TSA decreased this heterochromatin mark but also that TSA-promoted acetylation did not significantly affect H3K9 residues (Figure 5). One possible explanation is that acetylation might occur at a higher frequency on other H3 or H4 lysine residues. Taken together, our results suggest that the changes in HP1 localization are most likely due to the reduction in H3K9me3 after TSA treatment rather than to alterations in HP1 protein translation.

**Figure 6:**
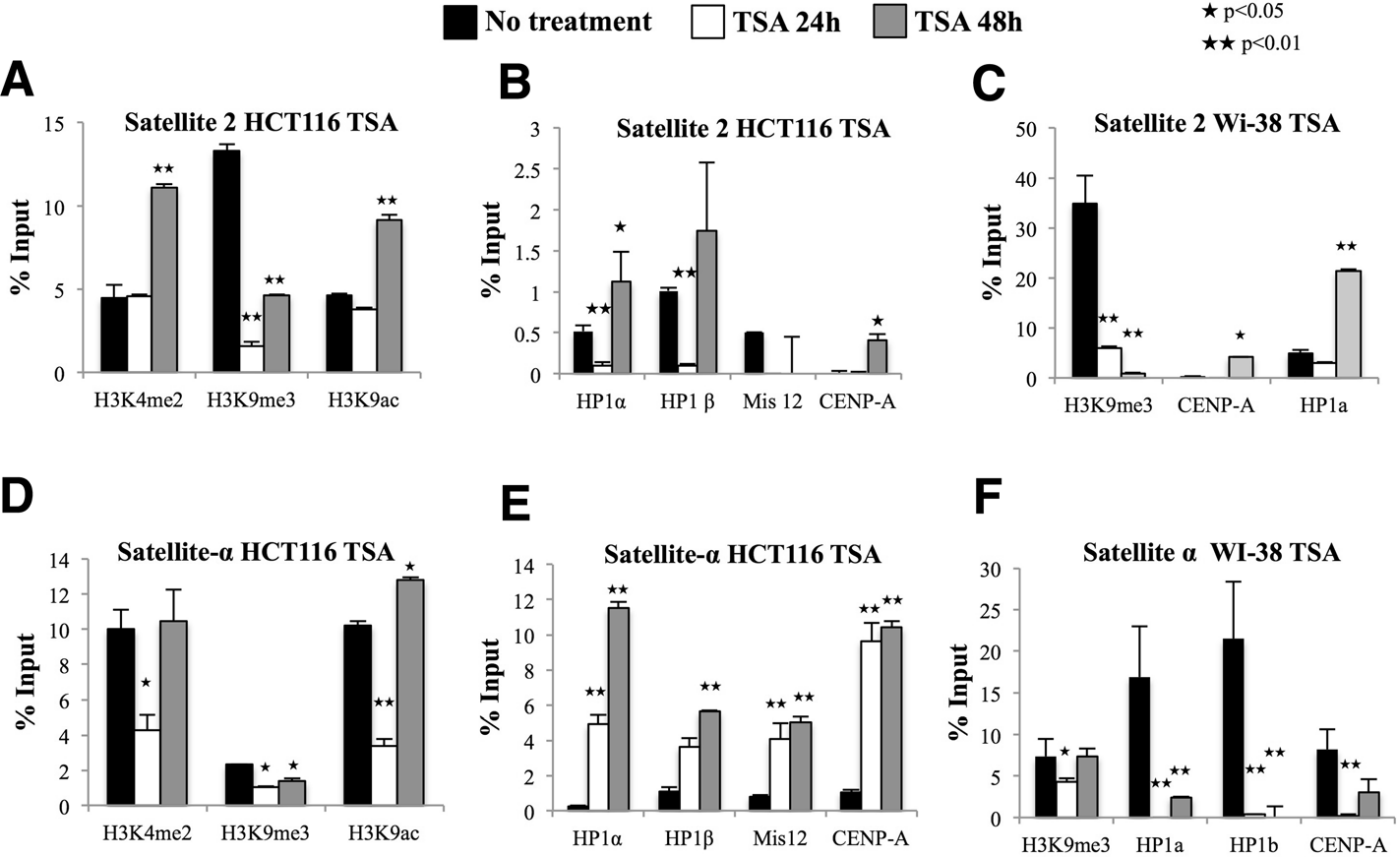
**Epigenetic changes and protein dynamics at satellite-2 and satellite-α regions after treatment with TSA for 24 and 48 h in HCT116 and WI-38 cells.** qRT-PCR analysis of the satellite-2 **(A-C)** and satellite-α **(D-F)** repeats was performed on DNA obtained from anti-H3K4me2, H3K9me3, H3K9ac, HP1α, HP1β, Mis12 and CENP-A ChIP assays in untreated cells (Black bar) and TSA-treated cells for 24 (white bar) or 48 h (gray bar). Normal rabbit IgG was employed as a negative control. ★p < 0.05 and ★★p < 0.01 indicate significant differences between treated and untreated cells, as evaluated by Student’s t-test.

TSA treatment leads to changes in the nuclear localization of HP1 proteins. Therefore, we performed a ChIP assay after TSA exposure in HCT116 cells. We found that TSA abolished the abundance of H3K9me3 at satellite-2 regions after the first 24 h of treatment; this abolishment was associated with the loss of HP1α and HP1β after 24 h of TSA exposure (Figure 6A-B). Surprisingly, HP1α and HP1β were reestablished at satellite-2 chromatin after 48 h of TSA exposure, even though H3K9me3 was dramatically diminished by the treatment (Figure 6A-B). H3K4me2 and H3K9ac were not significantly changed after 24 h of treatment, but were significantly increased after 48 h (Figure 6A), suggesting that TSA treatment promotes a significantly open chromatin state at the satellite-2 region after 48 h of exposure.

Satellite-α, in addition to satellite-2, regions exhibited losses in H3K4me2, H3K9me3, and H3K9ac after 24 h of TSA treatment. However, after 48 h, H3K4me2 was restored to the centromere, whereas H3K9ac was significantly increased (Figure 6D). Although H3K9me3 was significantly reduced by the TSA treatment, some fraction of this mark remained at the centromere (Figure 6D). After 24 h of treatment, CENP-A was increased by 10-fold at satellite-α chromatin and remained enriched by 10-fold after 48 h of TSA treatment (Figure 6E). As observed in the immunofluorescence experiments, HP1α and HP1β were enriched 6- and 4-fold, respectively, and this fold enrichment was proportional to the fold increase in Mis12 at satellite-α chromatin, suggesting that the presence of Mis12 was associated with the abundance of HP1 at the centromere (Figure 6E). We used the GAPDH gene promoter as a control to evaluate the effect of TSA treatment. Although we observed no increase in H3K9ac, we did observe a loss of H3K9me3 as a result of the treatment, suggesting that H3K9ac is not essential for GAPDH upregulation (data not shown).

Because HCT116 cells showed changes in histone marks and HP1 protein levels after TSA treatment, we next questioned whether the same modulation occurred in normal cells. Therefore, we treated WI-38 cells with TSA for 24 and 48 h and performed a ChIP assay using antibodies against H3K9me3, CENP-A, and HP1. TSA treatment for 24 h reduced H3K9me3 levels in satellite-2 regions and nearly abolished this histone mark after 48 h. Therefore, HP1α was reduced as its reader mark was diminished (Figure 6C). In contrast to the observations made in HCT116 cells, after 24 h of exposure to TSA in WI-38 cells, HP1α and HP1β were reduced at the satellite-α region and were not reestablished after 48 h, even though H3K9me3 levels were reestablished (Figure 6F). This result suggests that the co-localization observed by immunofluorescence was located at specific centromeric and pericentromeric chromatin regions and not at the chromosome 1 centromeric region.

## Discussion

### HP1α and HP1β localize at centromeric regions after TSA exposure in HCT116 cells, but their levels are reduced in WI-38 cells

In different cell lines and animal models, aberrant mitotic phenotypes have been attributed to a lack of pericentromeric H3K9me3, changes in H4K20me, and abnormal regulation of HDAC. CIN has been observed as increased chromosome misalignment in metaphase, nondisjunction in anaphase, and lagging chromosomes in telophase, and as high rates of aneuploidy and the appearance of micronuclei during cytokinesis or early G_1_ phase [17,22,23].

HP1 is essential, especially in the pericentromeric region, which is enriched with H3K9me3 and H4K20me3 modifications, hypoacetylated H3 and H4, and highly methylated regions along satellite repeats [4,24,25]. Little is known regarding the effects of HP1 during mitosis; however, the reduction of HP1 by TSA-promoted mitotic defects has been previously reported [18,20]. We observed that HP1α and HP1β, together with H3K9me3, are located in pericentromeric regions and that the centromeric localization of HP1 is preserved during mitosis, although HP1α and HP1β are dissociated in other regions of the chromosome. Likewise, we observed changes in HP1α and HP1β abundance throughout the cell cycle at the centromeric and pericentromeric chromatin of chromosome 1. This result was consistent with a previous report in which HP1 proteins underwent large-scale dissociation in G_2_-phase cells [26]. The change in HP1 localization during mitosis could also be attributed to the presence of acetylated histones on mitotic chromosomes, which decreases the accessibility of histone N-tails to the antibody, as was observed for H3 serine 10 phosphorylation [18,27].

Centromeres contain CENP-A nucleosomes interspersed with H3K9me2/3 nucleosomes but exhibit low levels of H3K4me2 enrichment [28,29]. In this regard, we observed clear HP1-enriched foci co-localizing with CENP-A and H3K9me3 during interphase; this colocalization continued throughout mitosis. In HCT116 cells, we found that H3K4me2 is reduced at centromeric chromatin during mitosis. It has been reported that H3K4me2 is an essential modification of centromeric chromatin that is required for its long-term maintenance and function, whereas the enrichment of H3K9me3 and H3K9ac fluctuate significantly throughout the cell cycle [22]. H3K9me3 has been reported to increase in abundance during G_2_/M in mammals, whereas H3K9me2 abundance remains constant during the cell cycle [22,30,31]. Our results are consistent with an increase in H3K9me3 at satellite-2 chromatin and at non-specific satellite-α regions; moreover, no significant increase was detected at satellite-α regions upon analyzing chromosome 1 during mitosis. Remarkably, these results suggest that H3K9me3 abundance at satellite-2 regions during mitosis is not equal in all cells, as was previously suggested.

Increasing evidence has shown that the KMN network in humans is a binding partner of HP1 and that HP1 may participate in recruiting and directing the Mis12 complex to the centromere during interphase by direct interaction with Mis14 [11,12,32]. In HCT116 cells, we observed that during interphase, HP1α, HP1β and Mis12 are present at centromeric chromatin, in agreement with previous reports. In contrast, during mitosis, Mis12 was not enriched at the same site, although HP1α and HP1β were also reduced. This could be explained by the nature of the Mis12 interaction with HP1: HP1α and Ndc80 are competitive binders of Mis12, suggesting that these proteins have identical or overlapping binding sites [13]. For the Ndc80 complex to localize to the kinetochore, it is necessary to displace most of the HP1α from Mis12. As a result, Mis12 and the Ndc80 complex play a role at the outer kinetochore but not at mitotic centromeric chromatin during metaphase.

### TSA induces differential changes in centromeric and pericentromeric chromatin and in CIN induction in HT116 and WI-38 cells

TSA treatment promotes histone hyperacetylation, which becomes visible at the nuclear periphery, as well as the reduction of many heterochromatin regions in the nucleus [20,33,34]. Due to this reduction of heterochromatin, we evaluated whether short-term TSA treatment modifies the centromeric and pericentromeric regions due to HP1 protein enrichment. We found that both HP1α and HP1β were enriched in foci that co-localized with H3K9me3 and CENP-A, suggesting that both HP1 proteins not only remained at pericentromeric heterochromatin but were also enriched at constitutive heterochromatin and expanded to centromeric chromatin. This result is similar to the findings of a study conducted in HeLa cells after short-term TSA treatments [20]. We also observed that H3K9me3 modifications and HP1 proteins are generally reduced in the nuclei after TSA treatment in HCT116 cells. Interestingly, this result is contradictory to a recent report in which HP1 protein localization to centromeric chromatin was reduced and scattered in the nucleus after the treatment of murine cell lines with low concentrations of TSA [35]. In this regard, it has been observed that upon inhibition of heterochromatin acetylation, HP1 disperses within the nucleus. Another report observed that HDAC inhibition caused the dynamic recruitment of HP1 proteins to pericentromeric chromatin in a primary human cell line, suggesting that HP1 mobilization after treatment could protect the kinetochore structure and function and that HP1 proteins behave differently in human and mouse cells [19,35-37].

TSA could influence histone acetylation at pericentromeric heterochromatin regions, as reported for low doses in other cell models, but requires several cell cycles to take effect [20]. In contrast, following short-term treatment with 1 μM TSA, we observed that HP1α and HP1β relocalized to centromeric chromatin, where H3K9me3, although reduced, was still present during interphase and mitosis in both normal and transformed cells. Our result is in contrast with the results of other reports indicating that, whereas H3K9ac was increased at the satellite-III region, the abundance of H3K9me2/me3 after treatment with TSA for 15 h TSA or with other HDACi treatments was not changed [19,38]. These results suggest that the chromatin at pericentromeric and centromeric regions responded differently to TSA treatment.

We observed that Mis12 localization appears to be intrinsically associated with HP1β during the cell cycle. Remarkably, this phenomenon did not occur in normal cells, and H3K9me3 levels were nearly unaffected by treatment, whereas both HP1 proteins, together with CENP-A, were reduced, suggesting that the mechanism that promotes HP1 protein localization to the centromere in HCT116 cells fails in WI-38 cells.

It has been suggested that the inhibition of histone deacetylation before mitosis is associated with improper chromosome condensation, which might induce mitotic checkpoint activation and CIN [18,20,39]. Such inhibition at the pericentromeric region might lead to deficient kinetochore assembly during mitosis [20,21,39]. However, such effects upon the kinetochore composition and microtubule dynamics were observed without an effect on Mis12 [39]. Although we agree that Mis12 was not globally affected, Mis12 was enriched at the centromeric chromatin of chromosome 1 after TSA exposure, indicating that the effect on Mis12 is more fine-tuned.

TSA treatment induced aneuploidy in both cell types. We observed that the cytotoxic effects of TSA were more pronounced in HCT116 cells than in WI-38 cells. Additionally, we found a significant percentage of tetraploid cells after TSA exposure in WI-38 cells. One reason for the cytotoxicity of TSA used at high doses is thought to be the disruption of two cell-cycle checkpoints: the G2 phase checkpoint and the mitotic spindle checkpoint [40,41]. This dual checkpoint disruption results in the premature exit of cells from mitosis, possibly followed by apoptosis; it has been reported in HeLa cells that treatment for 24 h with 1 μM TSA induced apoptosis in approximately 20% of cells [33]. Therefore, we suggest that this cell death is caused by aneuploidy because TSA decreases proliferation and promotes apoptotic cell death by inducing caspase 3/7 activity [42]. However, a recent report in murine fibroblastic NIH 3 T3 cells observed significant effects on the mitotic index and micronuclei induction only upon treatment of high concentrations of HDACi for 48 h [35]. A similar phenomenon might be occurring in WI-38 cells, wherein these cells might be more resistant to TSA than HCT116 cells.

**Figure 7:**
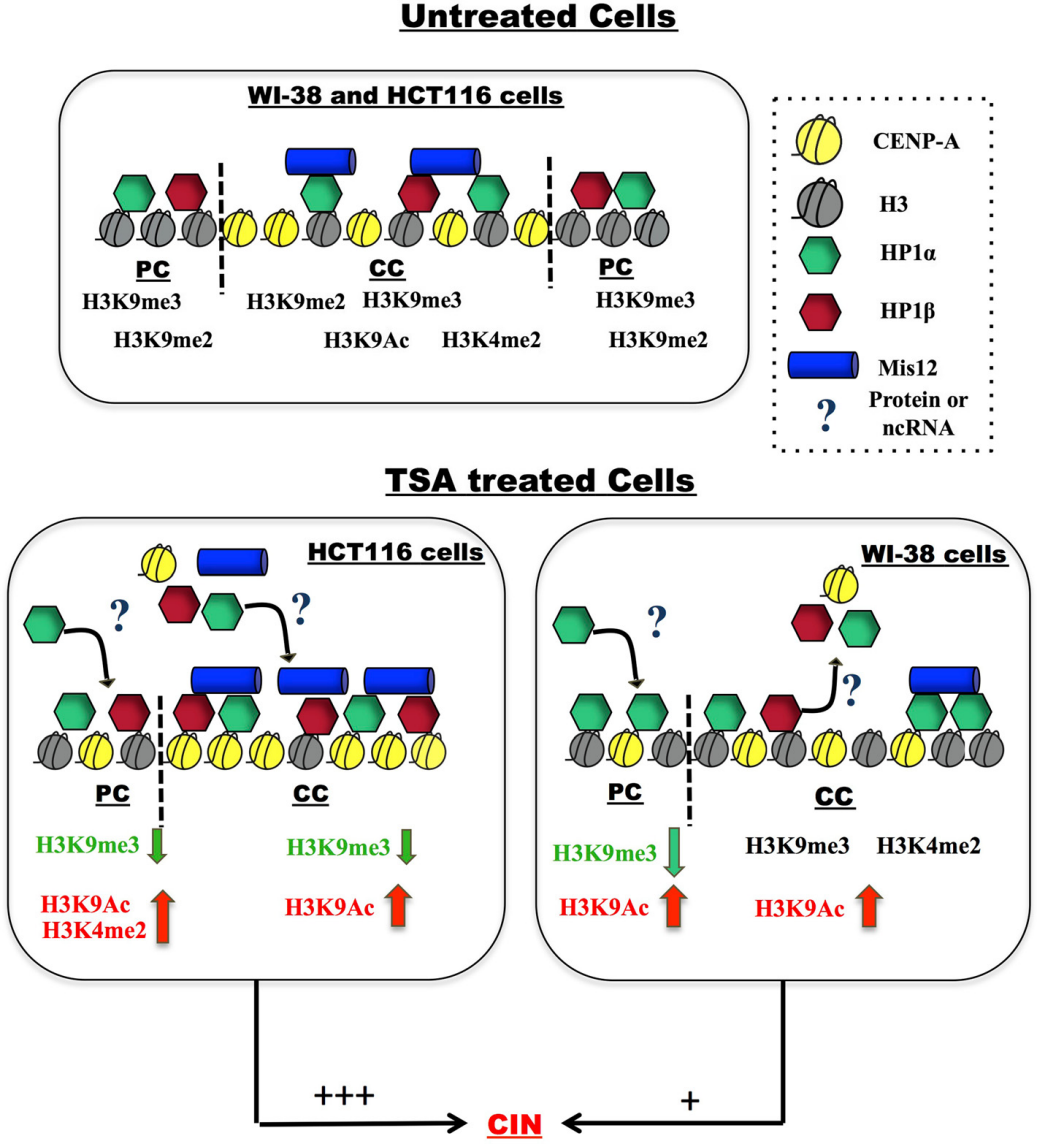
**The effect of TSA exposure on HP1α/β localization and centromeric and pericentromeric chromatin modifications, as well as on the relationship with CIN in WI-38 and HCT116 cells.** Upper panel: Untreated WI-38 and HCT116 cells presented a similar localization of HP1α and HP1β along centromeric (CC) and pericentromeric chromatin (PC). At PC, H3K9me2/3 were enriched together with HP1 proteins; at CC, CENP-A was enriched during mitosis, whereas H3K4me2, H3K9me2/3 and H3K9ac modifications, as well as HP1α and HP1β and Mis12 proteins, fluctuate through interphase and mitosis. Lower panel: After treatment of HCT116 cells with TSA, H3K9me3 was significantly reduced at PC and CC, resulting in increased H3K4me2 and H3K9ac at PC satellite-2 regions; HP1α/β were initially significantly reduced. However, they later recovered by an unknown mechanism that could include other proteins or could involve an ncRNA PC transcript [43-45]. CC was enriched with H3K9ac and CENP-A, together with HP1α/β and Mis12 proteins. HCT116 cells proliferated with low levels of cell arrest and exhibited CIN in 50% of the cells. In both WI-38 and HCT116 cells, PC presented reduced H3K9me3, whereas H3K9ac and HP1 were enriched; moreover, CC was depleted of CENP-A and HP1, and no significant reduction in H3K9me3 was observed, even though H3K9ac was increased. While CIN was still generated, it was reduced compared with HCT116 cells.

We show that differential changes in centromeric chromatin occur in HCT116 and WI-38 cells in response to TSA. Based on this result, together with the reduction of heterochromatin markers in pericentromeric chromatin regions, we propose the model shown in Figure 7, in which untreated HCT116 and WI-38 cells present a similar distribution of HP1 proteins and chromatin modifications at the centromeric and pericentromeric regions. However, after TSA treatment, H3K9me3 was reduced, whereas H3K9ac and CENP-A were increased at pericentromeric and centromeric chromatin in both cell lines. Furthermore, in HCT116 cells, HP1 proteins were recovered to the pericentromeric and centromeric regions regardless of the status of the reader mark. Suggesting that an unknown mechanism that could include other proteins or could involve an non-coding RNA transcript might be recruiting HP1 proteins [43-45]. In contrast, in WI-38 cells, the HP1 proteins were lost from centromeric chromatin after treatment. CIN occurred after TSA treatment, especially in HCT116 cells, in which very low levels of cell cycle arrest were promoted compared with those in WI-38 cells. These results suggest that the epigenetic landscape of centromeric and pericentromeric chromatin leads to the differential promotion of CIN upon TSA treatment in tumoral and non-tumoral cell lines.

## Conclusions

The data presented here provide new insight into the epigenetic landscape of centromeric chromatin, as well as into the role of HP1α and HP1β proteins in chromosome segregation and, by extension, cell division stability. We also present evidence of differences in the organization of centromeric chromatin and HP1 localization in response to TSA in the WI-38 and HCT116 cell lines. These differences are associated with CIN resulting from a chromatin disturbance caused by reduced H3K9me3 levels and TSA-induced hyperacetylation. The effects of TSA were substantially more pronounced in the malignant, transformed HCT116 cells than in WI-38 cells, leading to more significant chromosome mis-segregation and CIN. In addition, we believe that one cause underlying the effects of TSA-induced CIN and cell cycle arrest might be the deregulation of centromeric and pericentromeric chromatin regions, leading to the possibility that epigenetic regulation of the centromere might alter the response of tumor cell lines to TSA treatment. Nonetheless, many questions remain regarding the nature of centromere epigenetics, how these epigenetic modifications regulate kinetochore assembly and their role in chromosome segregation, as well as how communication is established between centromeric and pericentromeric chromatin.

## Materials and methods

### Antibodies

The following antibodies were used: anti-ACA (immunofluorescence (IF) dilution 1:200; Antibodies Incorporated, Davis, CA, USA 15-235-F); anti-Mis12 (C-13; IF dilution 1:80, ChIP 4 μg; Santa Cruz, Santa Cruz, CA, USA sc-107750); anti-H3K4me2 (IF dilution 1:200, ChIP 3 μg; Millipore Temecula, CA, USA 07-030); anti-H3K9ac (IF dilution 1:200, ChIP 2.4 μg; Abcam, Cambridge, MA, USA ab10812); anti-H3K9me2 (IF dilution 1:200, ChIP 3 μg; Abcam, ab1220), anti-H3K9me3 (WB dilution 1:250; Abcam, ab8898; IF dilution 1:200, ChIP 3 μg; Diagenode, Denville, NJ, USA CS-056-050); anti-CENP-A (IF dilution 1:200, ChIP 5 μg; Abcam, ab13939); anti-HP1α (IF dilution 1:100, ChIP 4 μg; Abcam, ab77256); anti-HP1β (IF dilution 1:100, ChIP 4 μg; Abcam, ab10811); anti-GFP (ChIP 4 μg; Abcam, ab290); and anti-H3 N-terminal (ChIP 1.5 μg; Sigma, St. Louis, MO, USA, H9289-200 μl).

### Cell viability (IC_50_) after TSA treatment

Human WI-38 and HCT116 cells were obtained from ATCC (CCL-75 and CCL-247). All cell lines were tested and authenticated and were maintained in Eagle’s Minimum Essential Medium (EMEM; ATCC) and McCoy (Gibco) medium, respectively, supplemented with 10% fetal bovine serum (Gibco) and antibiotics; the cells were incubated at 37°C in a 5% CO_2_ atmosphere. The cells were treated with TSA (Sigma, T8552-5MG) at 37°C for 24 and 48 h. IC_50_ concentrations were determined by plating 80,000 cells in 24-well dishes containing 0.5 ml of medium and incubating overnight at 37°C; TSA was added when cultures reached 80% confluence. Cells were washed with PBS and fixed with 70% ethanol at -20°C, then washed in PBS and stained with 1% crystal violet. After washing, the stain was solubilized in 33% acetic acid, and the absorbance was determined in an ELISA reader at 570 nm. The analyses were performed in triplicate in three independent experiments. The IC_50_ values were calculated by linear regression analysis of the dose-response data using the points in the exponential region of the curve. The concentrations used for the TSA experiments were below the IC_50_: 4.9 μM for HCT116 cells and 9.4 μM for WI-38 cells.

### Immunofluorescence

WI-38 and HCT116 cells were grown on 18-mm glass coverslips (PEARL 7201) with EMEM (ATCC) and McCoy medium (Gibco), respectively, supplemented with 10% fetal bovine serum (Gibco) and antibiotics, and the cells were incubated at 37°C in a 5% CO_2_ atmosphere. The cell lines were fixed with 2% paraformaldehyde (PFA) in 1X PBS (pH 7.4) for 10 min, followed by permeabilization in 0.4% IGEPAL (Sigma CA-630) in PBS for 10 min at room temperature and incubated with 0.5% BSA blocking buffer. For each pair of primary antibodies, the optimal order of addition was determined in preliminary experiments. With the exception of ACA, which was visualized with a fluorescein-conjugated secondary antibody, the fluorophores on the secondary antibodies were Alexa Fluor 488-conjugated (Invitrogen, Life Technologies, México; A11001 anti-mouse, A11008 anti-rabbit, and A11078 anti-goat) for green fluorescence and Cy3-conjugated (Millipore, Temecula, CA, USA; AP124C anti-mouse and AP1132C anti-rabbit) for red fluorescence. Following incubation with the primary and secondary antibodies, DNA was stained with DAPI. The cells were observed by fluorescence microscopy using a Zeiss Axio Imager A2 (Carl Zeiss®, Germany); the images were analyzed using the software AxioVision 4.8 (Carl Zeiss®, Germany). The cells were also observed by laser confocal microscopy using a Zeiss LSM 710 Duo (Carl Zeiss®, Germany); the images were analyzed using the Zen 2008 software (Carl Zeiss®, Germany).

### TSA treatment

Exponentially growing HCT116 and WI-38 cells were cultured on glass coverslips for 24 and 48 h in medium containing 1 μM/ml TSA (Sigma, T8552-5MG), with daily media changes. The cells were washed with PBS, fixed with PFA, and used for immunofluorescence analysis, as described above. For treated chromatin isolation, WI-38 and HCT116 cells were cultured on 100-mm culture plates and treated in the same manner as the cell cultures grown on glass coverslips. We used 1 μM TSA for 24 h and 48 h because at this concentration, we found a significant induction of CIN or centromeric chromatin remodeling. In addition, this concentration was below the IC_50_ for both cell lines.

### Electrophoresis and immunoblotting

After treatment with TSA, WI-38 and HCT116 cells were harvested in lysis buffer containing 50 mM Tris-HCl pH 7.5, 150 mM NaCl, 1% Nonidet P40, 0.5% deoxycholate, and the cOmplete Protease Inhibitors Cocktail (Roche) and were then sonicated. Then, 30 μg of protein was loaded onto a denaturing 10-20% gradient or 16% sodium dodecyl sulfate (SDS)-polyacrylamide gel and subsequently transferred to a nitrocellulose membrane. After incubation for 2 h in a PBS solution containing 5% albumin, the blots were exposed to the following primary antibodies: anti-HP1α (1:300); anti-HP1β (1:200); anti-H3 N-terminal (1:300); anti-H3K9me3 (1:250); anti-H3K9ac (1:250); and anti-CENP-A (1:200). The blots were incubated for 1 h at room temperature with the following horseradish peroxidase-conjugated secondary antibodies: goat anti-mouse IgG (1:10,000 Zymed); goat anti-rabbit IgG (1:15,000 Santa Cruz Biotechnology, Inc.); and chick anti-goat IgG (1:15,000 Chemicon International). The signal was subsequently detected by chemiluminescence (ECL kit from Millipore, USA) on Kodak X-Omat film. For the negative control, the primary antibody was omitted.

### Chromatin immunoprecipitation (ChIP)

The ChIP assay was performed using the OneDay ChIP kit (Diagenode, NJ, USA, Kch-onedIP-180), following the manufacturer’s instructions. For all experiments, at least two chromatin preparations from independent controls and TSA-treated cells were analyzed. To obtain mitotic and interphase cell chromatin, control and TSA-treated cells were exposed to 2 μg/ml nocodazole for 12 h; the mitotic cells were isolated by the shake-off method, and fluorescence-activated cell sorting (FACS) was used to select the population with 90% enrichment of mitotic cells. Using this method, the interphase cells remained on the culture plates and were harvested separately from the mitotic cells. Chromatin from each cell population was fixed with 1% formaldehyde, and the cells were counted to ensure that 1x10^6^ cells were used for each IP. The chromatin was then extracted, and ChIP was performed following the manufacturer’s instructions. As a negative control, we used a normal rabbit IgG antibody (sc-2027, Santa Cruz Biotechnology, USA).

The obtained results represent experiments from three separate amplifications that were used to calculate the standard deviation. To balance any difference in the amounts of ChIP products and input for qPCR, the amplification efficiency (AE) was calculated to within 10% of the input. The fold of the enrichment was calculated from the AE of specific experimental amplicons against the AE of the background IgG amplicon, which was amplified in triplicate by a fast optical 96-well qPCR reaction plate (Applied Biosystems). The qPCR reaction was performed using Thermo Maxima SYBR Green/ROX 1 PCR Master Mix (Thermo Scientific, K0222) with a StepOnePlus Real–Time PCR System (Applied Biosystems, 4376600). Total H3 immunoprecipitation was used to calibrate the increase in enrichment generated by chromosome duplication during mitosis.

### ChIP primers

The primers used for the ChIP qPCR analysis were as follows: 5′- TCGTTCCCAAAGTCCTCCTGTTTC-3′ (Fwd) and 5′-TCCGCAGCCGCCTGGTTC-3′ (Rev) for the GAPDH promoter; 5′-AGCCCTTCCCGCTCTTCTGTT-3′ (Fwd) and 5′-CGGCAGAGACGTAAGACTGGCAAA-3′ (Rev) for the WIF1 promoter; 5′-ATCGAATGGAAATGAAAGGAGTCA-3′ (Fwd) and 5′-GACCATTGGATGATTGCAGTCA-3′ (Rev) for human chromosome 1 juxtacentromeric satellite-2 (Abcam, ab85781); 5′-AAGGTCAATGGCAGAAAAGAA-3′ and 5′-CAACGAAGGCCACAAGATGTC-3′ (Abcam, ab85782) for human chromosome 1 centromeric satellite-α; and 5′-GAAGTTTCTGAGAATGCTTCTG-3′ (Fwd) and 5′-CTCACAGAGTTGAACCTTCC-3′ (Rev) for the satellite-α 175-bp monomers.

### Chromosome spread and counting

WI-38 and HCT116 cells were cultured on 22×22-mm glass coverslips until 70% confluence was reached; the cells were then treated with TSA for 24 or 48 h. The culture medium was removed and replaced with fresh medium after 24 h. The cells were treated for 3 h with 80 ng/ml colcemid (KaryoMAX GIBCO, USA 15210-040) to induce mitotic arrest and then incubated for 30 min at 37°C in hypotonic buffer (75 mM KCl) that had been pre-warmed to 37°C. The cells were fixed with three 2-minute washes in a 3:1 methanol:acetic acid solution and air-dried. The G banding standard protocol was performed with trypsin-Giemsa solution to stain mitotic chromosomes. For each condition, a certified cytogeneticist evaluated 50 metaphases in duplicate.

### Statistical analyses

Statistical significance was determined using Student’s t-test or one-way analysis of variance (ANOVA). All of the results are expressed as the mean ± SEM, and we used a significance value of p < 0.05. We performed Levene’s test to compare the significance of the control versus the 24- and 48-h TSA treatments or the 24- versus the 48-h TSA treatment, with a significance value of p < 0.05 in the metaphase counting analysis. Statistical analysis was performed using the GraphPad Prism 5 software®.

## Additional files

Additional file 1: Figure S1. Localization of H3K4me3, H3K9ac and Mis12 in WI-38 and HCT116 cells under basal conditions. **(A)** WI-38 cell fluorescent microscopy images of the localization of CENP-A with H3K4me2 (lane 1) and H3K9ac (lane 2). **(B)** Chromatin localization by fluorescent microscopy of CENP-A with H3K4me2 (lane 1) and H3K9ac (lane 2). DNA is marked with DAPI; the images show the most common distribution of the proteins after the analysis of 100 cells (%); the boxes represent a magnification of the immunofluorescence results; M, mitotic cell. Additional file 2: Figure S2. Chromosome immunoprecipitation controls for active and repressive epigenetic marks during interphase (black) and mitosis (gray). **(A)** The GAPDH gene promoter was analyzed for open chromatin state marks. **(B)** The WIF1 gene promoter was analyzed for repressive marks. **(C)** The epigenetic landscape was observed using changes to the satellite-α repeat consensus sequence during the cell cycle. Changes in the enrichment of H3K4me2, H3K9me2/3, H3K9ac, HP1α, HP1β, Mis12 and CENP-A are shown. ★p < 0.05, ★★p < 0.01 indicate significant differences between interphase and mitosis, as evaluated by Student’s t-test.

## Abbreviations

ACA: Anti-centromere antibody
CCAN: Constitutive centromere-associated network
CENP-A: Centromeric protein A
ChIP: Chromatin immunoprecipitation
CIN: Chromosome instability
GAPDH: Glyceraldehyde-3-phosphate dehydrogenase
HDAC: Histone deacetylase
HDACi: Histone deacetylase inhibitor
HP1: Heterochromatin protein 1
KL1: Katanin-like 1
KMN network: KL1, Mis12, and Ndc80 complex
Mis12: Minichromosome instability 12; MIND kinetochore complex component
Mis14: Minichromosome instability 14; kinetochore-associated protein NSL1
ncRNAs: Non-coding RNAs
Ndc80: Kinetochore protein NDC80 homolog
TSA: Trichostatin A
WIF1: WNT inhibitor factor 1
