# Supplementary figures and images for: Differential distribution of HP1 proteins after trichostatin a treatment influences chromosomal stability in HCT116 and WI-38 cells
Source: Cell Div. 2014 Dec 30;9:6. doi: 10.1186/s13008-014-0006-2 (PMC4343280; doi:10.1186/s13008-014-0006-2)

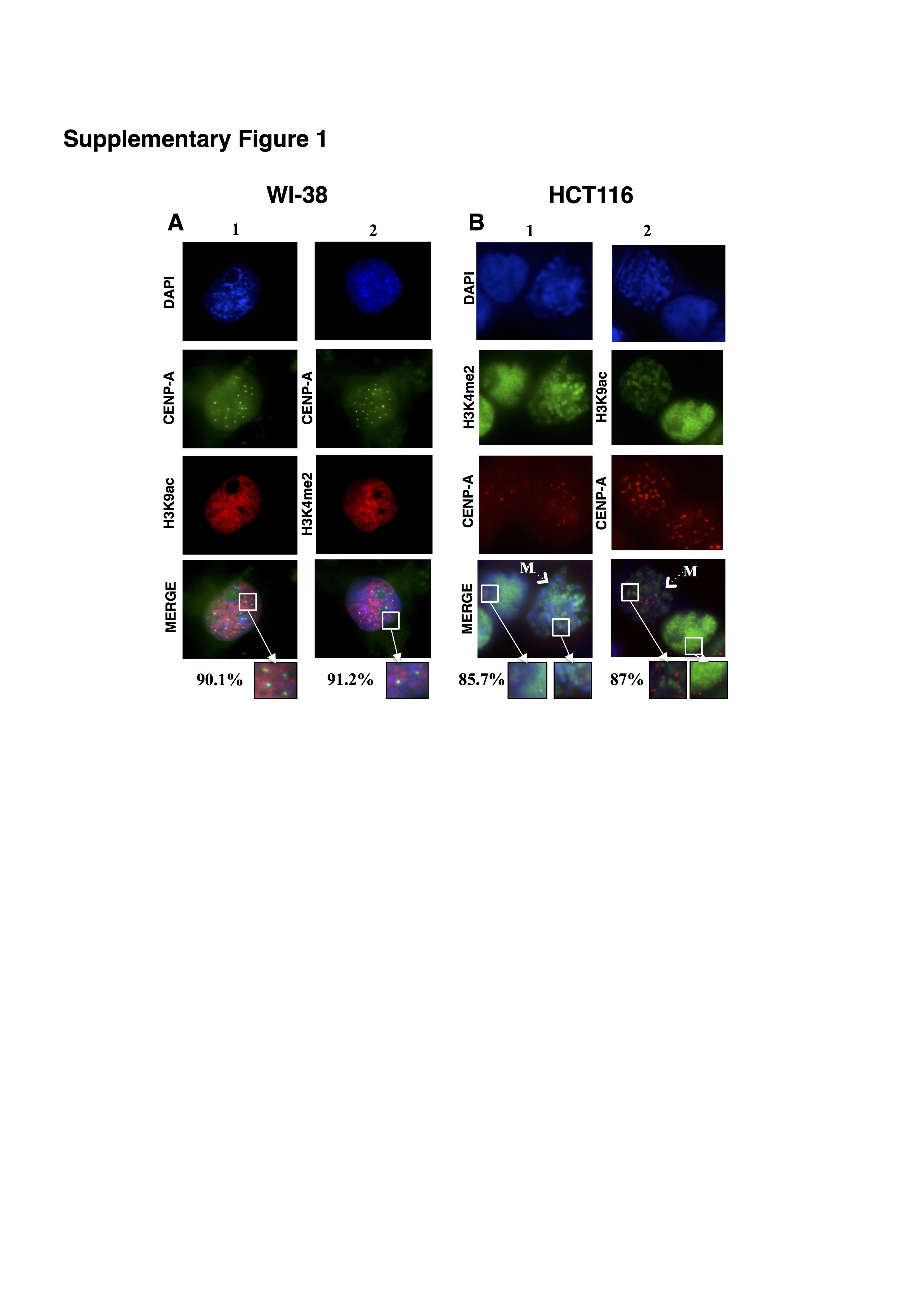

Supplement: Additional file 1: Figure S1. — Localization of H3K4me3, H3K9ac and Mis12 in WI-38 and HCT116 cells under basal conditions. (A) WI-38 cell fluorescent microscopy images of the localization of CENP-A with H3K4me2 (lane 1) and H3K9ac (lane 2). (B) Chromatin localization by fluorescent microscopy of CENP-A with H3K4me2 (lane 1) and H3K9ac (lane 2). DNA is marked with DAPI; the images show the most common distribution of the proteins after the analysis of 100 cells (%); the boxes represent a magnification of the immunofluorescence results; M, mitotic cell. [file 13008_2014_6_MOESM1_ESM.jpeg]

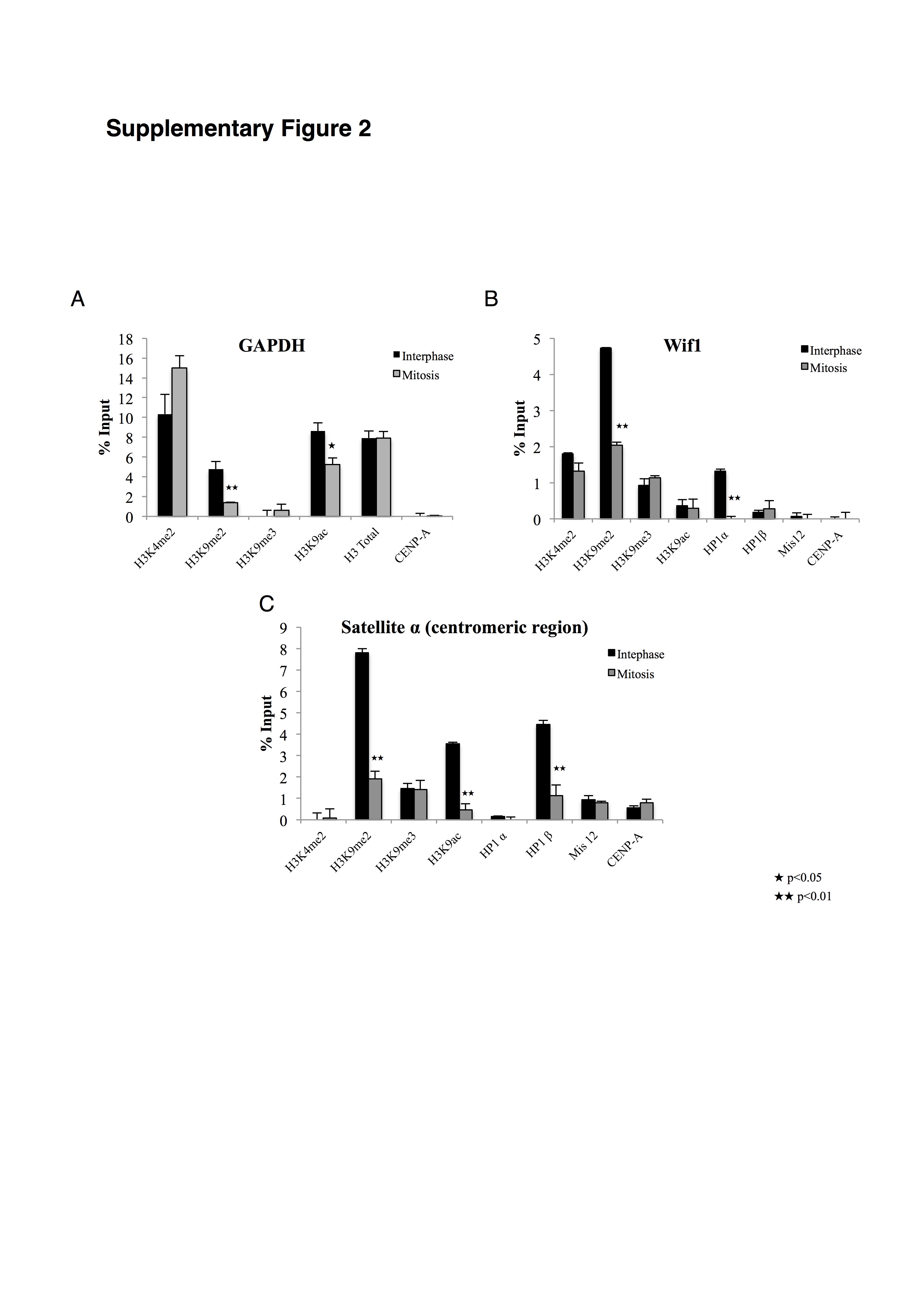

Supplement: Additional file 2: Figure S2. — Chromosome immunoprecipitation controls for active and repressive epigenetic marks during interphase (black) and mitosis (gray). (A) The GAPDH gene promoter was analyzed for open chromatin state marks. (B) The WIF1 gene promoter was analyzed for repressive marks. (C) The epigenetic landscape was observed using changes to the satellite-α repeat consensus sequence during the cell cycle. Changes in the enrichment of H3K4me2, H3K9me2/3, H3K9ac, HP1α, HP1β, Mis12 and CENP-A are shown. ★p < 0.05, ★★p < 0.01 indicate significant differences between interphase and mitosis, as evaluated by Student’s t-test. [file 13008_2014_6_MOESM2_ESM.jpeg]
